# Natriuretic peptides as novel regulators of dendritic cells-mediated inflammation

**DOI:** 10.1007/s00018-025-05769-8

**Published:** 2025-07-11

**Authors:** Giorgia Manni, Estevao Carlos Silva Barcelos, Doriana Ricciuti, Benedetta Pieroni, Marco Gargaro, Giulia Mencarelli, Hans Acha-Orbea, Vincenzo Nicola Talesa, Letizia Mezzasoma, Francesca Fallarino

**Affiliations:** 1 Department of Medicine and Surgery, University of Perugia, Perugia, Italy; 2 Department of Pharmaceutical Sciences, University of Perugia, Perugia, Italy; 3 Department of Biochemistry CIIL, University of Lausanne, Epalinges, Switzerland

**Keywords:** Cardiac hormones, Antigen presenting cells, Autoimmune diseases, Inflammatory diseases, Natriuretic peptide receptor antagonist, Cytokines

## Abstract

Natriuretic Peptides (NPs), including atrial (ANP) and brain (BNP) types, exert pleiotropic effects in regulating immune responses via the Natriuretic Peptide Receptor-1 (NPR1), expressed across various immune cells. While NPs are established inhibitors of inflammasome activation and IL-1β secretion in human monocytes, their role in dendritic cells (DCs)-key regulators of innate and adaptive immunity-remains unclear. Inflammasome activation in DCs can yield both protective and detrimental outcomes depending on the context of the disease, suggesting that modulating this pathway could offer a promising pharmacological strategy for controlling immune responses. This study explored the regulation of the NLRP3 inflammasome by NPs in two conventional DC subsets: cDC1 and cDC2. We found that both subsets express basal levels of the NPR1 receptor, which increase under inflammatory conditions. Additionally, cDCs themselves produce ANP and BNP during inflammation. Although both subsets express basal levels of NLRP3 inflammasome proteins, cDC2 display a more robust NLRP3/IL-1β activation in response to LPS + ATP stimulation compared to cDC1. Notably, the NPs/NPR1 axis suppresses NLRP3 activation more effectively in the cDC2 subset by acting at translational and post-translational levels. These findings highlight NPs as a novel mechanism for controlling the inflammatory phenotype of cDCs and underscores NPs/NPR1 axis as therapeutic target for immune modulation of DC subsets.

**Graphical abstract:**

**Figure:**
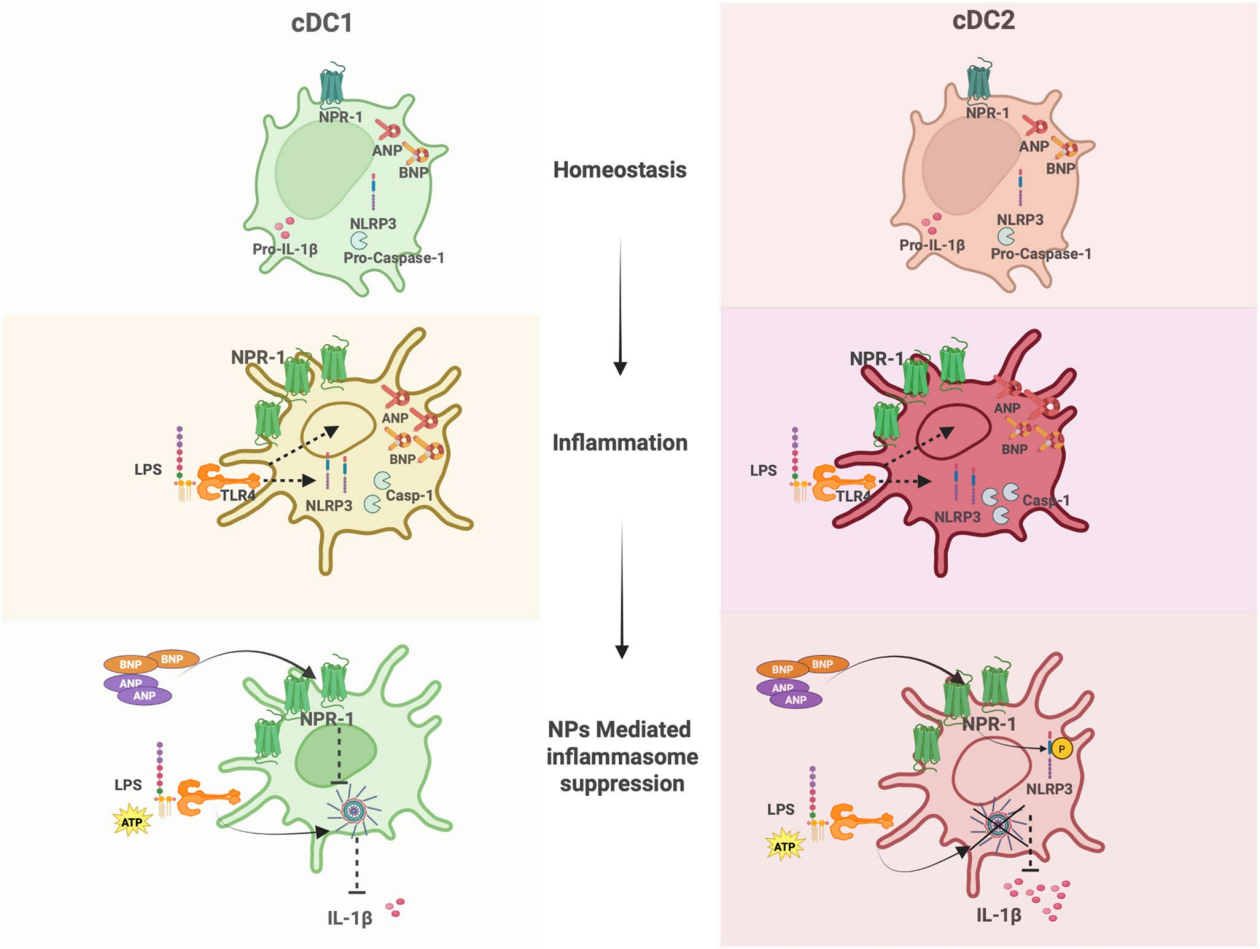
cDC1 and cDC2 are equipped with the ANP/BNP–NPR1 axis and are sensitive to NLRP3 inflammasome activation. The natriuretic peptides ANP and BNP, through NPR1, represent a novel immunoregulatory mechanism that more effectively suppresses the inflammatory response in cDC2s than in cDC1. The NPs/NPR1 axis emerges as a potential therapeutic target for the control of pathological inflammation. Created in BioRender. https://BioRender.com/nrcr3m9

## Introduction

Natriuretic Peptides (NPs), including Atrial Natriuretic Peptide (ANP) and B-type Natriuretic Peptide (BNP), are cardiac hormones involved in fluid and cardiovascular homeostasis. By binding to Natriuretic Peptide Receptor-1 (NPR1), they activate cGMP pathways, influencing phosphodiesterases, protein kinases (PKG-I and -II), and ion channels [1, 2]. While primarily known for their cardiovascular roles, growing evidence highlights the NPs/NPR1 axis in regulating inflammation and immune responses [2–5]. NPs and NPR1 are expressed in immune cells, with their activity modulated by inflammatory factors [3, 6]. Elevated plasma NPs are observed in conditions like sepsis [7], COVID-19 [8], and autoimmune diseases [9], indicating that inflammation triggers NPs release [10]. Preclinical models show NPs exert anti-inflammatory effects [11–14], with circulating NPs protecting against visceral adipose inflammation and insulin resistance [2]. However, NPR1-deficient mice show reduced pulmonary and allergic inflammation, suggesting also a pro-inflammatory role for NPs in specific conditions [15]. In macrophages, NPs modulate cytokines such as Tumor Necrosis Factor (TNF)-α, nitric oxide (NO), interleukin-(IL)−10 (IL-10), and IL-1beta (IL-1β) secretion in both tumor and non-tumor cells [3, 16, 17].

IL-1β is a key pro-inflammatory cytokine involved in inflammation, immune responses, and processes such as angiogenesis, fibrosis, and tumor progression [17]. It plays a critical role in innate immunity, promoting the acute phase response and the recruitment of inflammatory cells [18]. To prevent host tissue damage, IL-1β production is tightly regulated through transcriptional and post-translational mechanisms. Innate immune receptor signaling up-regulates pro-IL-1β expression, while inflammasome-mediated Caspase-1 activation leads to pro-IL-1β maturation leading to IL-1β production [19].

Inflammasomes are cytoplasmic supramolecular complexes activated in response to cellular perturbations triggered by infection and sterile injury. Inflammasomes are crucial for host defense, detecting pathogen-associated molecular patterns (PAMPs) and danger-associated molecular patterns (DAMPs) via nucleotide-binding oligomerization domain (NOD)-like receptors (NLRs) and absent in melanoma 2 (AIM2)-like receptors (ALRs). The NLR pyrin domain containing 3 (NLRP3) inflammasome is the most well-characterized in this family. Notably, the NLRP3 inflammasome plays a central role in immune defense against infections, with activation occurring via a two-step mechanism. Priming signals induce transcriptional up-regulation of NLRP3, pro-IL-1β, and pro-IL-18, primarily through nuclear factor kappa-light-chain-enhancer of activated B cells (NF-kB) activation. Activation signals then lead to inflammasome oligomerization through post-translational modifications like phosphorylation and ubiquitination [20]. Upon activation, NLRP3 recruits pro-Caspase-1 through the adaptor apoptosis-associated speck-like protein containing a Caspase-recruitment domain (ASC), triggering inflammasome assembly and Caspase-1 activation, which then matures IL-1β and IL-18 [19].

Dysregulation of NLRP3/IL-1β pathway has been associated to the pathogenesis of a wide range of disorders including Crohn’s diseases, cryopyrin-associated periodic syndromes, psoriasis, neurological diseases, diabetes, gout, silicosis, cardio-metabolic disorders, atherosclerosis, COVID-19 and cancer [21–23].

Although a remarkable number of knowledges described the NLRP3 inflammasome functions in immune regulation, less is known about its role in DCs, the main actors and orchestrators of innate and adaptive immunity.

DCs are professional antigen presenting cells that drive T cells activation and polarization leading to different types of immune responses, but also immune regulation or immune tolerance. Both human and murine DCs are heterogeneous cells consisting of two main cell populations, the plasmacytoid DCs (pDCs) and conventional DCs (cDCs) that in turn are divided into cDC1 and cDC2 [24]. The different DC subsets are specialized to induce specific T cell responses exhibiting different patterns of inflammasome activation [25]. However, the exact role of inflammasome activation in DCs and in the generation of antigen T cell responses is poorly understood. Although inflammasome activation typically leads to IL-1β secretion and is often accompanied by pyroptosis, certain cells, like DCs, can remain alive and persist in a hyperactive state [26]. Furthermore, studies have shown that the hyperactive murine cDC1 subset can stimulate robust cytotoxic CD8^+^ T-cell responses, contributing to anti-tumor activity and resulting in tumor regression [27]. In contrast, inflammasome activation without pyroptosis in the human cDC2 subset not only promotes the secretion of IL-1β but also induces the release of IL-12 family cytokines. This leads to strong Th1 and Th17 CD4^+^ T-cell responses, which may play a critical role in driving inflammatory and autoimmune diseases [25]. In addition, recent findings reveal that both murine cDC1 and cDC2 actively suppress pyroptotic inflammasome activation through the transcription factors IRF8 and IRF4, respectively. [27]. Despite the growing interest in inflammasome activation in cDCs, the factors involved in its regulation are not yet fully understood. NPs have been shown to modulate inflammasome activation and expression in various immune cells. Human DCs express NPR1 [28], however, the role of the NP/NPR1 axis in these cells remains poorly explored.

This study aimed to characterize the expression profiles of the NLRP3 inflammasome and the NPs/NPR1 axis in cDC1 and cDC2 subsets. Additionally, it sought to investigate the contributions of ANP and BNP to the NLRP3/IL-1β response during cDCs stimulation with LPS and ATP.

## Materials and methods

### Reagents

All the chemicals used in the present study were analytical grade reagents from various sources. Human ANP was obtained from Merck KGaA (#A1663, Darmstadt, Germany) and dissolved in 5% acetic acid. Human BNP (# 011–03, Phoenix Europe GmbH, Germany) was dissolved in H_2_O. LPS (from Escherichia coli 0111: B4, (#L2630), and ATP (#A2383) were obtained from Merck KGaA (Darmstadt, Germany) and were dissolved in H_2_O). The NPR-A antagonist A71915(Arg⁶,β-cyclohexyl-Ala⁸,D-Tic^1^⁶,Arg^1^⁷,Cys^1^⁸)-Atrial Natriuretic Factor (6–18)-amide) (H-3048) was from Bachem (#4030385, Bubendorf, Switzerland). All the primary antibodies, unless otherwise stated, were from Cell Signalling Technology (Leyden, The Netherlands).

### Cell culture and drug treatments

MutuDC_1940_ (cDC1) and mutuCD4^−^DC2 (cDC2) cells were cultured in complete culture medium: IMDM + GlutaMAX^TM^ Supplement (#31980, GIBCO), 10 mM HEPES (#15630, GIBCO), 0.075% NaHCO3, 50 μM β-mercaptoethanol (#31350, GIBCO), 8% heat inactivated FBS (#10270–106, GIBCO), 50U/mL penicillin, 50 μg/mL streptomycin (#15070, GIBCO) at 37 °C in a humidified incubator with 5% CO2 [29]. Cells were harvested by treatment with a non-enzymatic cell dissociation buffer (5 mM EDTA, 20 mM HEPES in PBS). Cells were seeded at density of 5 × 10^5^ cells/well in 48-well culture dishes. After 24 h, medium was replaced and cells were pre-treated for 10 min with human ANP or BNP (0.1 μM), exposed to LPS (100 ng/ml) for 1 h and then to ATP (5 mM) for 30 min. In independent experiments, treatments were extended for 24 h and 10 min before adding ATP (5 mM) for the last 30 min, ANP and BNP treatment was repeated. In independent experiments, H-3048 (10 µM) was added to cells 10 min before treatments. Acetic acid (0.0005% assay concentration) produces no significant toxicity; therefore, all the relative treatments were compared to this latter control.

At the end of the treatments, total cell lysates were prepared using RIPA buffer with protease and phosphatase inhibitors.

### Measurements of secreted IL-1β

Measurements of secreted IL-1β were performed in cell supernatant after cDCs exposure to the indicated compounds for 24 h at 37 °C. After treatments, supernatants were collected and human IL-1β levels were determined by the specific ELISA kit, according to the manufacturer's guidelines (Thermo Fisher Scientific # 88–7013-88).

### Western blot analysis

Total proteins (15 µg) were separated by 12% sodium dodecyl sulfate–polyacrylamide gel electrophoresis (SDS-PAGE) and transferred to nitrocellulose membrane. Non-specific binding sites were blocked in Roti-Block (#A151.3 Roth GmbH, Germany) for 1 h at room temperature. The membranes were blotted overnight at 4 °C with the following Abs diluted 1:1000 in Roti-Block: anti-NLRP3 (D4D8 T) (#15101 T), anti-Cleaved Caspase-1 (E2G2I) (#89,332), rabbit monoclonal Abs (mAb), anti-NPR1 (#PA5-29,049), anti-ANP (#PA5-29,559), anti-BNP (#PA5-21,321), anti-phospho-NLRP3 (Ser 295) (#PA5-105,071) rabbit polyclonal Abs (pAb) (ThermoFisher Scientific, Rockford, USA). After washing with TBST, blots were incubated for 1 h at room temperature with the appropriated HRP-conjugated secondary Abs (1:5000 dilution) and revealed using the enhanced chemi-luminescence (ECL) system (#WBKLS0500, Merck KGaA, Darmstadt, Germany). Membranes were stripped and re-probed with anti-α-tubulin (#T8328 Sigma-Aldrich) as loading control. Densitometric analyses were performed with ImageJ software (https://imagej.nih.gov/ij/).

### RNA extraction, cDNA synthesis and real time PCR

Mutu dendritic cells, cDC1 and cDC2, were cultured at a density of 0.5 × 10^6^/ml as previously described. After stimulation, cells were lysed in TRIzol® (#15,596,026, ThermoFisher) and RNA was isolated according to manufacturer’s protocol. cDNA was transcribed using Quantitect Reverse transcription kit (#205,313, Qiagen) according to manufacturer’s protocol. Gene expression analysis was performed by Real-time PCR using iTaq Universal SYBR Green Master Mix (#1,725,124, Bio-rad) for mouse *Nlrp3*, *Caspase-1*, *IL1b*, *Anp*, *Bnp*, *Npr1* and *b-Actin*, reported in Table 1, with the Mx3000P qPCR System (Stratagene). Each sample was normalized to *b-Actin* and values were determined by the relative quantification method (ΔΔCT) (mean ± SD of triplicate determinations). Data are presented as normalized transcript expression in the samples relative to normalized transcript expression in control cultures (in which fold change = 1).

**Table 1:** List of primers used in the study

*Gene*	*Primers Sequence*
*b-Actin*	Fw: GGC TCC TAG CAC CAT GAA GA Rev: AGC TCA GTA ACA GTC CGC C
*Nlrp3*	Fw: TGG ATG GGT TTG CTG GGA T Rev: CTG CGT GTA GCG ACT GTT GAG
*Caspase-1*	Fw: TTG AAA GAC AAG CCC AAG GTC Rev: CTG GTG TTG AAG AGC AGA AAG C
*IL1-b*	Fw: ACC TTC CAG GAT GAG GAC ATG A Rev: AAG GTC ACA CAC CAG CAG GTT A
*Nppa*	Fw: TTG GAG CCC AGA GTG GAC TA Rev: AGT GGC AAT GTG ACC AAG CT
*Nppb*	Fw: AAG ATG CAG AAG CTG CTG GA Rev: GCC TTG AGA CCG AAG GAC TC
*Npr1*	Fw: GTG TGA ACC GGA AAC GCA TT Rev: ACC GAA ACA TCC AGT CCA GG

### Data source and transcriptomic analysis

Microarray data were obtained from the Gene Expression Omnibus (GEO) repository under accession number GSE203450. After log2 transformation, the absolute expression was normalized across the arrays. Differential expression between groups was analyzed using the"voom"function from the"limma"package. Genes were considered differentially expressed if they had an adjusted p-value (FDR) of less than 0.05 and a logFC of ± 0.25. Volcano plots were created using the"ggplot2"and"ggrepel"packages, while heatmaps were constructed based on gene z-scores using the “ComplexHeatmap” package. All analyses were conducted in the R environment.

### Statistical analysis

Results were expressed as means ± SD of at least three independent experiments performed in triplicate. The statistical significance of differences between treated and untreated cells was assessed by one-way ANOVA, two-way ANOVA or Student's t-test. Differences between groups were considered significant when *P < *0.05.

## Results

### NPs/NPR1 profile expression in cDCs

**Fig. 1:**
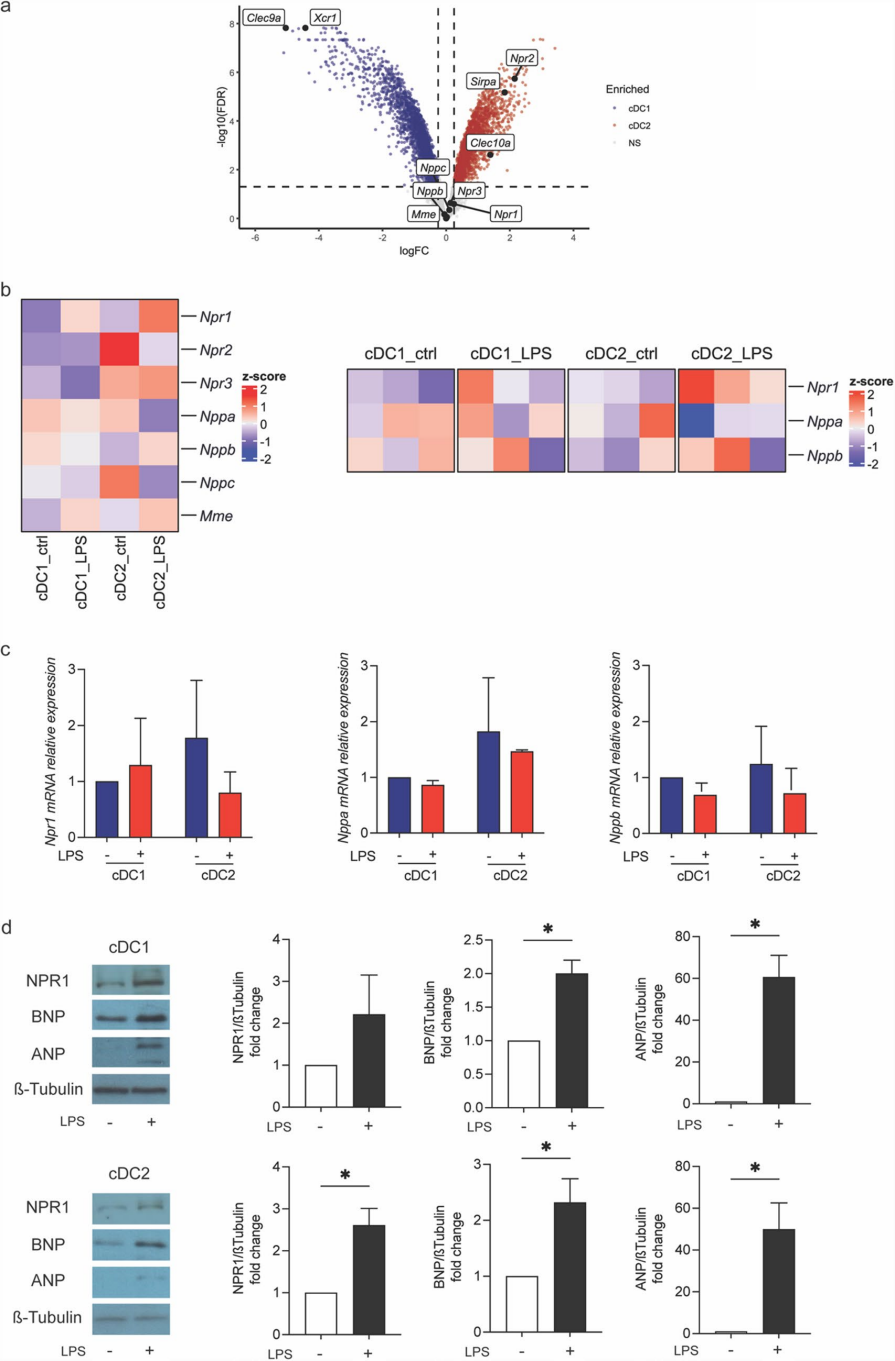
Natriuretic Peptide Axis profile expression in cDC subsets. **a** The volcano plot shows differentially expressed genes between steady-state cDC1 and cDC2 derived from bone marrow cells, highlighted by colors (enriched in cDC1, blue; enriched in cDC2, red; not significantly enriched, gray). **b** The heatmap shows the expression of z-scores of genes related to NPs axis in bone marrow derived cDC1 and cDC2 under steady-state condition and after stimulation with LPS for 24 h. **c**, **d** Mutu cDC1 and cDC2 cells were cultured for 24 h. Medium was replaced, and cells were treated with LPS (100ng/ml for 24 h). ANP, BNP and NPR1 gene **c** and protein **d** expression were assessed by quantitative RT-PCR and WB analysis, respectively. The values were normalized to β-tubulin. Data are represented as mean ± SD, with *n = *3. Untreated cells were used as control and assumed as 1; *p < *0.05 *vs* untreated cells by unpaired t-test

A previous study demonstrated that human pDCs express NPR1 [28], highlighting the potential involvement of the NP/NPR1 axis in immune regulation. To investigate the role of ANP and BNP in modulating NLRP3 inflammasome activation in cDCs, we examined the NPs/NPR1 expression profile in murine cDC1 and cDC2 subsets. Microarray data (GSE203450) [30], were re-analyzed for genes critical to the NPs axis, including *Npr1*, *Nppa*, and *Nppb*. No significant differences were observed in the mRNA expression levels of *Nppa*, *Nppb*, or the receptor gene *Npr1* in purified bone marrow-derived cDC1 and cDC2 under steady-state conditions, as illustrated by the Volcano plot (Fig. 1a). Key genes of the NPs/NPR1 axis from the same dataset were further analysed and showed as heatmaps comparing cDC1 and cDC2 both at steady state (control) and in inflammatory conditions (i.e., following 24 h LPS treatment). The data showed that LPS stimulation induced *Npr1* mRNA expression more prominently in cDC2 than in cDC1, while *Nppa* and *Nppb* expression remained relatively unchanged (Fig. 1b).

We next assessed *Npr1*, *Nppa*, and *Nppb* gene and protein expression in mutu cDC1 and cDC2 cell lines. Real-time PCR confirmed that both mutu cDC1 and cDC2 expressed the *Npr1* receptor, *Nppa*, and *Nppb* transcripts under steady-state conditions, with no significant changes in response to LPS stimulation (Fig. 1c). At the protein level, both mutu cDC1 and cDC2 were found to express the NPR1 receptor, and a slight increase in its expression was observed after 24 h LPS stimulation particularly in cDC2. Notably, ANP levels showed a marked increase upon LPS stimulation in both types of cDCs, while BNP protein levels exhibited a modest increase upon LPS stimulation in both cDCs (Fig. 1d).

These results demonstrate that cDC1 and cDC2 are sources of both ANP and BNP under inflammatory conditions, accompanied by a slight increase in NPR1 receptor expression. This suggests that these endogenous peptides may regulate cDCs functions through both endocrine, but also autocrine loops.

### cDC1 and cDC2 cells display differential inflammasome activation

Previous studies have demonstrated that NPs can modulate inflammasome activation in myeloid cells, particularly in human monocytes [5]. However, inflammasome expression and activation in DCs remains uncharacterized. To address this, we re-analyzed, as shown in Fig. 1a, the murine dataset of bone marrow derived cDCs [30], to assess the expression of inflammasome-related genes in the two main cDC subsets under steady-state conditions and following LPS stimulation.

**Fig. 2:**
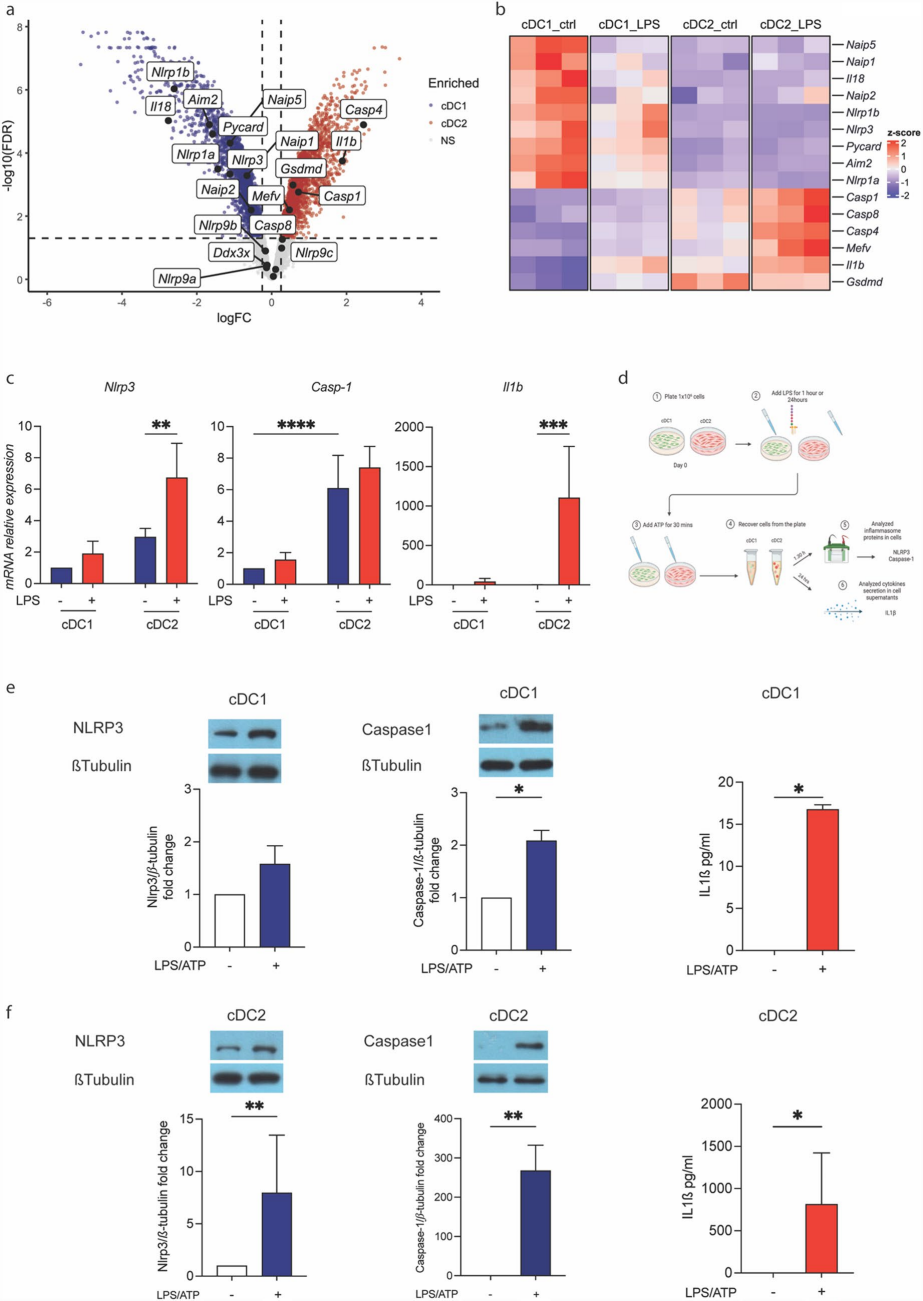
Differential NLRP3-inflammasome activation in cDC subsets. **a** The volcano plot shows differentially expressed steady-state genes between cDC1 and cDC2 derived from bone marrow cultures. Key inflammasome genes differentially expressed were highlighted by colors (enriched in cDC1, blue; enriched in cDC2, red; not significantly enriched, gray). **b** The heatmap shows the expression of z-scores for genes related to inflammasome in bone marrow derived cDC1 and cDC2 under steady-state conditions and after stimulation with LPS for 24 h. **c** Mutu cDC1 and cDC2 cells were cultured for 24 h. Medium was replaced, and cells were treated with LPS (100ng/ml for 24 h). Nlrp3, Caspase-1 and Il-1β gene expression were assessed by quantitative RT-PCR. **d** Schematic representation of inflammasome activation and analysis in cDC subsets (Created by BioRender.com). cDC1 and cDC2 cells were grown for 24 h. Medium was replaced, and cells were stimulated with LPS (100ng/ml for 60 min) and ATP (5 mM for 30 min) (i.e.LPS + ATP) **e**–**f**. NLRP3 and Caspase-1 protein expression levels in cDC1 **e** and cDC2 **f** were assessed by WB and normalized to β-tubulin. IL-1β protein secretion was measured by ELISA in culture supernatants in cDC1 **e** and cDC2 **f**. Data are represented as mean ± SD, with *n = *3;*p < 0.05 *vs* untreated cells by unpaired t-test; Data are represented as mean ± SD, with *n = *3. Untreated cells were used as control and assumed as 1; *p < 0.05; **p < 0.01; ***p < 0.001; ****p < 0.0001 *vs* untreated cells by unpaired t-test or by two-way ANOVA

Analysis of inflammasome-related genes, visualized using a volcano plot, revealed their expression in both cDC1 and cDC2 subsets (Fig. 2a). Notably, under steady-state conditions, mRNA transcripts encoding sensor proteins (e.g., *Aim2, Nlrp1a, Nlrp3, Pycard,* and others) were more abundant in cDC1, whereas those encoding effector proteins (e.g., *Casp1, Casp4, Il1b,* and *Gsdmd*) were enriched in cDC2 (Fig. 2a, b). To further assess how these transcripts respond to inflammatory conditions, we analyzed key inflammasome-related genes from the same dataset and visualized their expression as heatmaps. We found that LPS treatment induced expression of effector proteins in cDC2, but not in cDC1, and not significant transcriptional induction in sensor proteins in both cDCs subsets. Transcripts encoding sensors proteins appeared to be slightly downregulated in cDC1 upon 24 h LPS stimulation (Fig. 2b).

To further validate these findings, mutu cDCs were either left untreated or stimulated with LPS for 24 h, followed by analysis of *Nlrp3, Casp1,* and *Il1b* expression using real-time PCR. At steady state, mutu cDC1 and cDC2 exhibited comparable levels of these inflammasome-related genes. However, LPS stimulation for 24 h led to a significant increase in *Nlrp3* and *Il1b* expression, predominantly in the mutu cDC2 subset (Fig. 2c). Next, mutu cDC1 and cDC2 were stimulated with LPS and ATP (LPS + ATP), a classical trigger of NLRP3 inflammasome activation in macrophages, and inflammasome activation was assessed as outlined in Fig. 2d. LPS + ATP treatment resulted in differential activation of the inflammasome pathway between the two cDC subsets. Specifically, cDC2 cells exhibited robust inflammasome activation, as indicated by elevated protein levels of the NLRP3 receptor and the active p20 fragment of Caspase-1, compared to cDC1 cells. In line with these results, LPS + ATP stimulation led to higher IL-1β secretion in cDC2 cells compared to cDC1 cells (Fig. 2e, f).

Collectively, these findings indicate that the cDC2 subset is highly equipped to respond to inflammatory stimuli and exhibits a markedly stronger NLRP3 inflammasome activation upon LPS + ATP treatment compared to cDC1.

### NPs/NPR1 axis repress inflammasome activation in cDCs

**Fig. 3:**
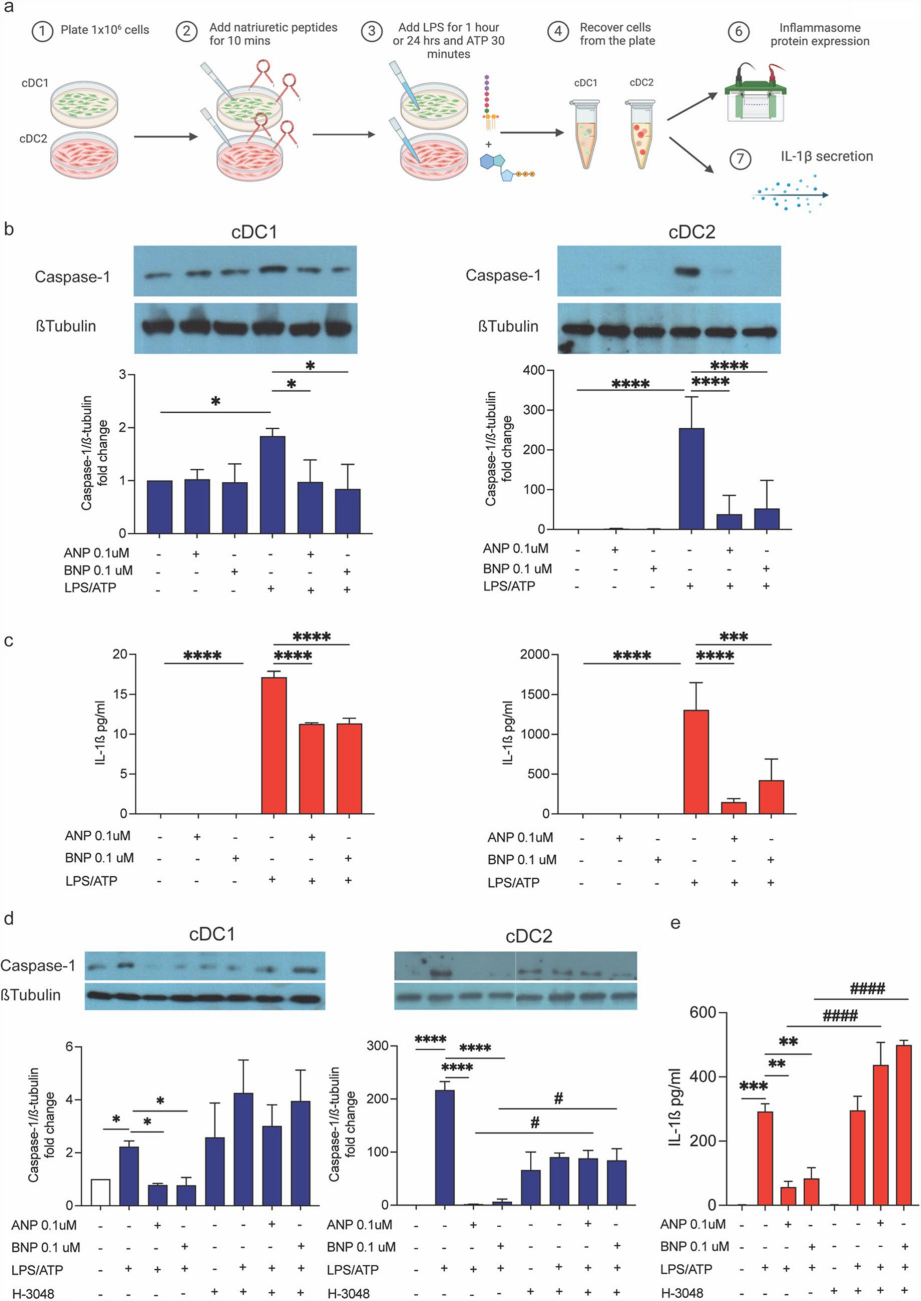
NPs/NPR1 axis repress NLRP3-Inflammasome activation in cDCs. **a** Graphical representation of the experimental model (Created by BioRender.com). cDC1 and cDC2 cells were grown for 24 h, medium was replaced, and cells were treated with ANP and BNP (0.1μM for 10 min) and then stimulated with LPS (100ng/ml for 60 min) and ATP (5 mM for 30 min) (LPS + ATP) and Caspase-1 protein expression was assessed by WB analysis **b** and the level of IL-1β in culture supernatants was determined by ELISA **c**. cDC1 and cDC2 cells were pre-treated with H-3048 (10μM for 10 min) before (LPS + ATP) stimulation and Caspase-1 activation **d** or IL-1β secretion **e** in culture supernatants were assessed by WB or ELISA, respectively. Untreated cells were used as control and assumed as 1, *n = *3; *p < 0.05; **p < 0.01; ***p < 0.001; ****p < 0.0001 *vs* untreated cells; ^#^*p < *0.05; ^# # # #^*p < *0.0001; LPS + ATP + ANP or BNP *vs* H-3048 + LPS + ATP + ANP or BNP treated cells; by one-way ANOVA followed by Tukey’s multiple comparisons test

Based on previous findings [5, 6, 31] and the observed expression of inflammasome-related genes and proteins in cDCs (Fig. 2a, b), we hypothesized that exogenous NPs might influence LPS + ATP-induced inflammasome activation in these cells. To test this, cDC1 and cDC2 cells were stimulated in vitro, and inflammasome activation was assessed as shown in Fig. 3a. Notably, brief exposure of cDCs to ANP or BNP (e.g. 10 min prior to LPS + ATP stimulation) effectively inhibited early Caspase-1 activation and significantly reduced IL-1β secretion at 24 h in both cDC1 and cDC2 subsets (Fig. 3b, c). This effect was more pronounced in cDC2 cells compared to cDC1, as expected (Fig. 3b, c).

To determine whether the NPs/NPR1 axis played a role in this inhibitory effect, we pretreated cDCs with H-3048, a potent NPR1 antagonist, before ANP or BNP exposure and subsequent LPS + ATP stimulation. H-3048 strongly reversed the NPs-mediated inhibition of LPS + ATP-induced Caspase-1 activation in both subsets (Fig. 3d). Interestingly, in cDC2 cells, H-3048 pretreatment also increased constitutive Caspase-1 activation (Fig. 3d). Regarding IL-1β secretion, H-3048 fully abrogated the inhibitory effect of NPs in cDC2 cells (Fig. 3e).

Overall, these findings demonstrate that the NPs/NPR1 axis suppresses inflammasome activation in cDCs, with a more pronounced inhibitory effect in cDC2 cells.

### NPs/NPR1 axis repress priming and oligomerization of NLRP3-Inflammasome platform in cDCs

**Fig. 4:**
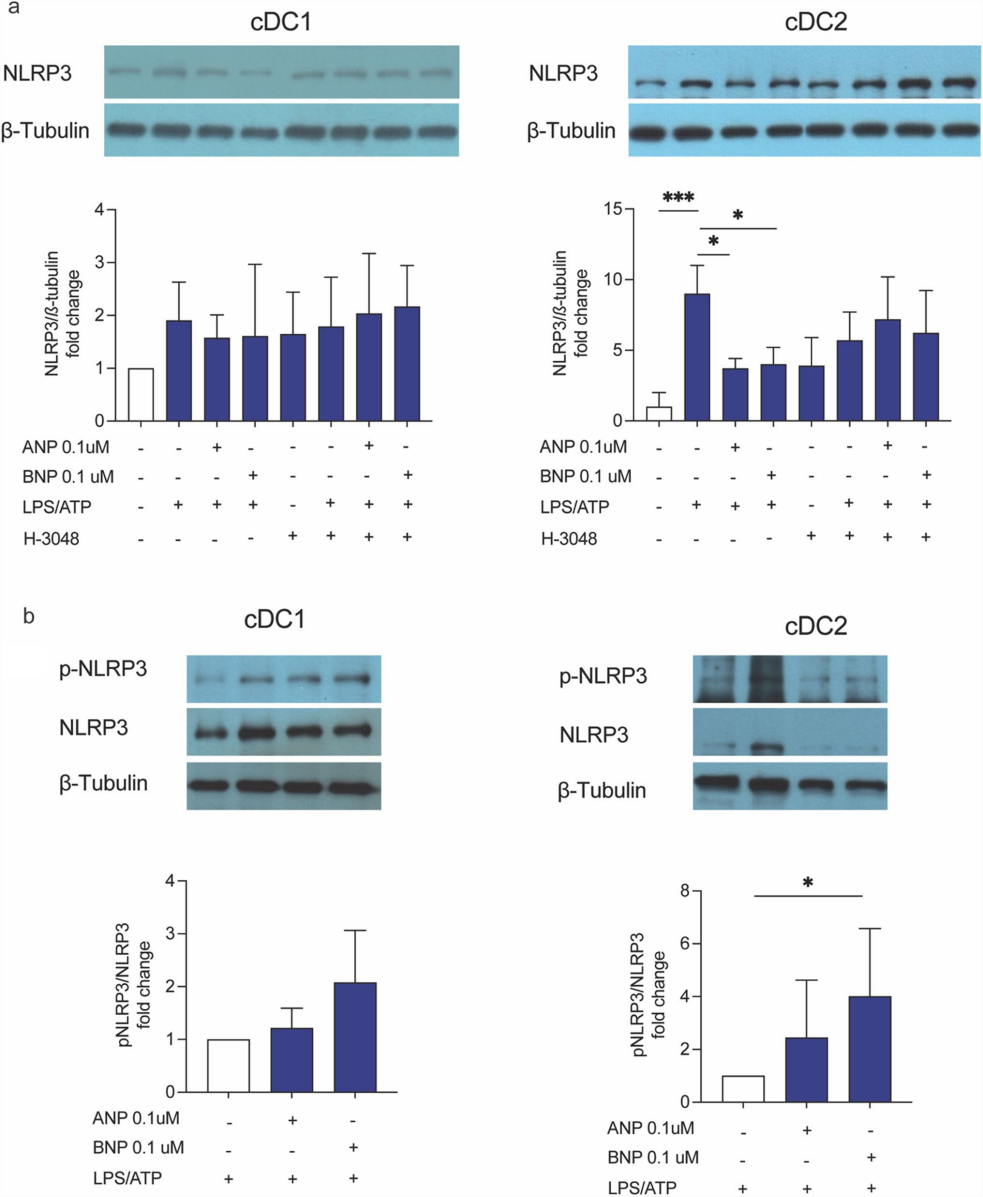
NPs/NPR1 axis represses priming and oligomerization of NLRP3-Inflammasome platform in cDCs. cDC1 and cDC2 cells were grown for 24 h. Medium was replaced, and cells were treated with ANP and BNP (0.1μM for 10 min) and then stimulated with LPS (100ng/ml for 60 min) and ATP (5 mM for 30 min) (LPS + ATP) in absence or presence of H-3048 pretreatment (10μM for 10 min). NLRP3 **a** and phospho-NLRP3 (Ser295) (p-NLRP3) **b** protein expression was assessed by WB analysis. For NLRP3 analysis untreated cells were used as control and assumed as 1; for p-NLRP3 analysis LPS + ATP cells were used as control and assumed as 1, *n = *3; *p < 0.05; ***p < 0.001 versus untreated or LPS + ATP stimulated cells by one-way ANOVA followed by Tukey’s multiple comparisons test

Canonical NLRP3 activation is a tightly regulated process that occurs in two distinct steps. The first step, considered rate-limiting, involves the upregulation of NLRP3 protein levels [32], while the second step includes inflammasome oligomerization, driven by post-translational modifications including NLRP3 phosphorylation [20, 21]. We found that both ANP and BNP reduced LPS + ATP-induced NLRP3 expression in cDC2 cells, an effect that was completely reversed by H-3048 (Fig. 4a). A similar trend was observed in cDC1 cells, although the changes were not statistically significant (Fig. 4a).

To further investigate whether NPs modulate NLRP3 activation through post-translational mechanisms, we assessed the effect of ANP and BNP on NLRP3 phosphorylation levels in LPS + ATP-treated cDC1 and cDC2 cells. Notably, we found that pre-treatment with ANP and BNP enhanced NLRP3 phosphorylation (Fig. 4b).

These findings indicate that the NPs/NPR1 axis negatively impact on both the priming and oligomerization steps of inflammasome activation in cDCs, highlighting its role as a new key checkpoint pathway, particularly in the cDC2 subset.

## Discussion

ANP and BNP are traditionally known for their roles in cardiovascular homeostasis and fluid balance. Beyond these classical functions, growing evidence has highlighted their involvement in modulating immune responses. Previous studies have demonstrated that both these peptides modulate inflammation in various immune cells and exert a strong inhibitory action on inflammasome activation in monocytes and macrophages [5, 6, 31]. However, their specific role in DCs, which are pivotal in bridging innate and adaptive immunity, remains largely unexplored. Existing literature indicated that plasmacytoid DCs express NPR1, suggesting a potential but uncharacterized involvement of the NPs/NPR1 axis in DCs-mediated immune regulation [28]. This study offers novel and compelling evidence that NPs, through their interaction with NPR1, serve as regulators of DCs inflammatory responses. By inhibiting NLRP3 inflammasome activation at multiple levels, NPs/NPR1 axis plays a crucial role in controlling DCs-driven inflammation, offering new insights in innate immune mechanisms modulation. We demonstrated, for the first time, that both cDC1 and cDC2 subsets not only express NPR1 but also produce ANP and BNP under inflammatory conditions, suggesting the existence of an autocrine regulatory loop that modulates DCs functions. Specifically, LPS stimulation was shown to increase NPR1 receptor and NPs protein expression in both cDC1 and cDC2, with a more pronounced increase in ANP than BNP, indicating an active role of endogenous NPs in shaping the inflammatory response of DCs.

A second remarkable observation is the differential inflammasome activation between cDC1 and cDC2 subsets, demonstrating that cDC2 populations are strongly equipped and respond to danger stimulation triggering a much more effective NLRP3 inflammasome activation and IL-1β secretion, compared to cDC1. As professional antigen-presenting cells, cDCs serve as a critical link between the innate and adaptive immune systems. NLRP3 inflammasome, on the other hand, acts as a master regulator of both systems by triggering the release of the pro-inflammatory cytokines IL-1β and IL-18 which are essential for the activation of adaptive immune cells [25, 33]. This renders the crosstalk between cDCs and the NLRP3 inflammasome crucial for the immune system's ability to detect and respond to infections and injury. The differential responsiveness of cDC1 and cDC2 cells to LPS + ATP stimulation, emphasizes the diverse roles of the two subsets in the inflammatory scenario where the exact role of inflammasome activation is yet poorly understood.

Importantly, we show that the NPs exert a significant inhibitory effect on inflammasome activation, with ANP and BNP treatment markedly reducing Caspase-1 activation and IL-1β secretion in both cDC subsets. This effect was specifically mediated through the NPR1 receptor, as demonstrated by using the NPR1 antagonist H-3048, that induced an increase in the constitutive active Caspase-1 in cDC2 cells. This effect highlights that cDC2 cells exhibit a stronger sensitivity to NPs-mediated regulation and suggests that the NPs anti-inflammatory effect may be especially effective when inflammation is more pronounced. Accordingly, elevated levels of NPs are observed in patients with critical non-cardiac diseases marked by significant immune system activation, such as sepsis, septic shock [7], COVID-19 [8], and autoimmune diseases [34]. Inflammasome activation in DCs can have either protective or detrimental effects, depending on the nature of the stimuli. In experimental murine models of autoimmune diseases, including glomerulonephritis [35], myocarditis [36], encephalomyelitis [37], graft-versus-host disease [38], and inflammatory bowel disease [39], inhibiting or knocking out the NLRP3 inflammasome in DCs led to an improvement in disease outcomes, associated with a shift in DCs towards a more tolerogenic/regulatory phenotype [39]. Therefore, the newly discovered potent inhibitory action of NPs/NPR1-axis on IL-1β secretion in DCs suggest that the targeting of NPs/NPR1 axis could be a promising therapeutic approach for controlling excessive inflammation in autoimmune diseases and chronic inflammatory conditions.

At the mechanistic level, we found that the NPs/NPR1 axis regulates inflammasome activity by targeting NLRP3 inflammasome activation at multiple levels. Specifically, pre-treatment with ANP and BNP both decreased NLRP3 protein expression and strongly enhanced NLRP3 phosphorylation at S295 in cDC2 subset. The level of NLRP3 protein is considered the rate-limiting factor in the initial step of inflammasome activation [32], while the second step involves the oligomerization of the platform through post-translational modifications. Among the post translational modifications, accumulating evidence points to the critical role of phosphorylation/dephosphorylation cycles both in NLRP3 inflammasome assembly and destabilization [20]. Accordingly, NPs-mediated cGMP/PKG-I axis activation dampens inflammasome platform assembly through a direct phosphorylation of NLRP3 at Ser295 also in monocytic cells [5]. Our data suggest that NPs regulate DCs inflammasome activity by dual action targeting both the priming and the oligomerization stages of NLRP3 platform activation.

An important consideration is whether DCs in specific pathological conditions retain the ability to respond to NPs or if disease-associated alterations, such as receptor downregulation or desensitization, affect their responsiveness. Moreover, the physiological levels of circulating NPs may vary significantly across different conditions, raising the question of whether endogenous concentrations are sufficient to exert immunoregulatory effects on DCs or if local production or administration is required. Finally, while our findings in murine models suggest a role for NPs in controlling the inflammatory phenotype of conventional DCs, further validation in human subsets will be important and addressed in future research, including the analysis of NPs receptor expression and signaling in human DCs.

In conclusion, this study provides compelling evidence that NPs serve as novel regulators of DCs inflammatory responses via the NPR1 receptor, offering a novel perspective on how innate immune mechanisms can be modulated to maintain immune homeostasis. These insights pave the way for future research to explore the therapeutic potential of modulating the NPs/NPR1 axis in inflammatory and autoimmune diseases where DC subsets play important roles.

## Data Availability

All data are available in the main text.

## References

[1] Potter LR, Yoder AR, Flora DR, Antos LK, Dickey DM (2009) Natriuretic peptides: their structures, receptors, physiologic functions and therapeutic applications. Handb Exp Pharmacol 341–366. 10.1007/978-3-540-68964-5_15 10.1007/978-3-540-68964-5_15 PMC4855512 19089336

[2] Goetze JP, Bruneau BG, Ramos HR, Ogawa T, de Bold MK, de Bold AJ (2020) Cardiac natriuretic peptides. Nat Rev Cardiol 17:698–717. 10.1038/s41569-020-0381-0 32444692 10.1038/s41569-020-0381-0

[3] Ogawa T, de Bold AJ (2012) Brain natriuretic peptide production and secretion in inflammation. J Transplant 2012:962347. 10.1155/2012/962347 23251786 10.1155/2012/962347 PMC3515950

[4] Zhang J, Li M, Yang Y, Yan Y, Li J, Qu J, Wang J (2015) NPR-A: A therapeutic target in inflammation and cancer. Crit Rev Eukaryot Gene Expr 25:41–46. 10.1615/CritRevEukaryotGeneExpr.2015012447 25955817 10.1615/critreveukaryotgeneexpr.2015012447

[5] Mezzasoma L, Talesa VN, Romani R, Bellezza I (2020) ANP and BNP exert anti-inflammatory action via NPR1/cGMP axis by interfering with canonical, non-canonical, and alternative routes of inflammasome activation in human THP1 cells. Int J Mol Sci 22.10.3390/ijms22010024 10.3390/ijms22010024 PMC7792787 33375031

[6] Mezzasoma L, Antognelli C, Talesa VN (2016) Atrial natriuretic peptide down-regulates LPS/ATP-mediated IL-1β release by inhibiting NF-kB, NLRP3 inflammasome, and caspase-1 activation in THP-1 cells. Immunol Res 64:303–312. 10.1007/s12026-0158751-0 26616294 10.1007/s12026-015-8751-0

[7] Vallabhajosyula S, Wang Z, Murad MH, Vallabhajosyula S, Sundaragiri PR, Kashani K, Miller WL, Jaffe AS (2020) Natriuretic peptides to predict short-term mortality in patients with sepsis: a systematic review and meta-analysis. Mayo Clin Proc Innov Qual Outcomes 4:50–64. 10.1016/j.mayocpiqo.2019.10.008 32055771 10.1016/j.mayocpiqo.2019.10.008 PMC7011015

[8] Gordon JS, Drazner MH (2021) Biomarkers of cardiac stress and cytokine release syndrome in COVID-19: a review. Curr Heart Fail Rep 18:163–168. 10.1007/s11897-021-00505-2 33666855 10.1007/s11897-021-00505-2 PMC7932899

[9] Avouac J, Meune C, Chenevier-Gobeaux C, Dieudé P, Borderie D, Lefevre G, Kahan A, Allanore Y (2014) Inflammation and disease activity are associated with high circulating cardiac markers in rheumatoid arthritis independently of traditional cardiovascular risk factors. J Rheumatol 41:248–255. 10.3899/jrheum.130713 24334650 10.3899/jrheum.130713

[10] Fish-Trotter H, Ferguson JF, Patel N, Arora P, Allen NB, Bachmann KN, Daniels LB, Reilly MP, Lima JAC, Wang TJ, Gupta DK (2020) Inflammation and circulating natriuretic peptide levels. Circ Heart Fail 13:e006570. 10.1161/CIRCHEARTFAILURE.119.006570 32507024 10.1161/CIRCHEARTFAILURE.119.006570 PMC7375923

[11] Kuhn M (2012) Endothelial actions of atrial and B-type natriuretic peptides. Br J Pharmacol 166:522–531. 10.1111/j.1476-5381.2012.01827.x 22220582 10.1111/j.1476-5381.2012.01827.x PMC3417485

[12] Song Z, Zhao X, Liu M, Jin H, Wang L, Hou M, Gao Y (2015) Recombinant human brain natriuretic peptide attenuates trauma-/haemorrhagic shock-induced acute lung injury through inhibiting oxidative stress and the NF-κB-dependent inflammatory/MMP-9 pathway. Int J Exp Pathol 96:406–413. 10.1111/iep.12160 26852688 10.1111/iep.12160 PMC4744823

[13] Yang H, Song Z, Jin H, Cui Y, Hou M, Gao Y (2014) Protective effect of rhBNP on intestinal injury in the canine models of sepsis. Int Immunopharmacol 19:262–266. 10.1016/j.intimp.2014.01.023 24508538 10.1016/j.intimp.2014.01.023

[14] Zhu N, Li T, Bai Y, Sun J, Guo J, Yuan H, Shan Z (2024) Targeting myocardial inflammation: investigating the therapeutic potential of atrial natriuretic peptide in atrial fibrosis. Mol Biol Rep 51:506. 10.1007/s11033-024-09393-w 38622341 10.1007/s11033-024-09393-w PMC11018689

[15] Wang X, Xu W, Mohapatra S, Kong X, Li X, Lockey RF, Mohapatra SS (2008) Prevention of airway inflammation with topical cream containing imiquimod and small interfering RNA for natriuretic peptide receptor. Genet Vaccines Ther 6:7. 10.1186/1479-0556-6-7 18279512 10.1186/1479-0556-6-7 PMC2291050

[16] Chiurchiù V, Izzi V, D’Aquilio F, Carotenuto F, Di Nardo P, Baldini PM (2008) Brain natriuretic peptide (BNP) regulates the production of inflammatory mediators in human THP-1 macrophages. Regul Pept 148:26–32. 10.1016/j.regpep.2008.02.009 18410972 10.1016/j.regpep.2008.02.009

[17] Mezzasoma L, Talesa VN, Costanzi E, Bellezza I (2021) Natriuretic peptides regulate prostate cells inflammatory behavior: Potential novel anticancer agents for prostate cancer. Biomolecules 11:794. 10.3390/biom11060794 34070682 10.3390/biom11060794 PMC8228623

[18] Dinarello CA (2009) Immunological and inflammatory functions of the interleukin-1 family. Annu Rev Immunol 27:519–550. 10.1146/annurev.immunol.021908.132612 19302047 10.1146/annurev.immunol.021908.132612

[19] Martinon F, Tschopp J (2007) Inflammatory caspases and inflammasomes: master switches of inflammation. Cell Death Differ 14:10–22. 10.1038/sj.cdd.4402038 16977329 10.1038/sj.cdd.4402038

[20] Kelley N, Jeltema D, Duan Y, He Y (2019) The NLRP3 inflammasome: An overview of mechanisms of activation and regulation. Int J Mol Sci 20:3328. 10.3390/ijms20133328 31284572 10.3390/ijms20133328 PMC6651423

[21] Guo H, Callaway JB, Ting JP-Y (2015) Inflammasomes: mechanism of action, role in disease, and therapeutics. Nat Med 21:677–687. 10.1038/nm.3893 26121197 10.1038/nm.3893 PMC4519035

[22] Yin M, Marrone L, Peace CG, O’Neill LAJ (2023) NLRP3, the inflammasome and COVID-19 infection. QJM Mon J Assoc Physicians 116:502–507. 10.1093/qjmed/hcad011 10.1093/qjmed/hcad011 PMC10382191 36661317

[23] Wang H, Ma L, Su W, Liu Y, Xie N, Liu J (2025) NLRP3 inflammasome in health and disease (Review). Int J Mol Med 55:48. 10.3892/ijmm.2025.5489 39930811 10.3892/ijmm.2025.5489 PMC11781521

[24] Eisenbarth SC (2019) Dendritic cell subsets in T cell programming: location dictates function. Nat Rev Immunol 19:89–103. 10.1038/s41577-018-0088-1 30464294 10.1038/s41577-018-0088-1 PMC7755085

[25] Hatscher L, Amon L, Heger L, Dudziak D (2021) Inflammasomes in dendritic cells: friend or foe? Immunol Lett 234:16–32. 10.1016/j.imlet.2021.04.002 33848562 10.1016/j.imlet.2021.04.002

[26] Zanoni I, Tan Y, Di Gioia M, Springstead JR, Kagan JC (2017) By capturing inflammatory lipids released from dying cells, the receptor CD14 induces inflammasome-dependent phagocyte hyperactivation. Immunity 47:697-709.e3. 10.1016/j.immuni.2017.09.015 29045901 10.1016/j.immuni.2017.09.010 PMC5747599

[27] Zhivaki D, Borriello F, Chow OA, Doran B, Fleming I, Theisen DJ, Pallis P, Shalek AK, Sokol CL, Zanoni I, Kagan JC (2020) Inflammasomes within hyperactive murine dendritic cells stimulate long-lived T cell-mediated anti-tumor immunity. Cell Rep 33:108381. 10.1016/j.celrep.2020.108381 33207188 10.1016/j.celrep.2020.108381 PMC7727444

[28] Morita R, Fujita T, Uchiyama T, Hori T (2009) Human plasmacytoid dendritic cells express an atrial natriuretic peptide receptor, guanylyl cyclase-A. Microbiol Immunol 53:403–411. 10.1111/j.1348-0421.2009.00133.x 19563399 10.1111/j.1348-0421.2009.00149.x

[29] Fuertes Marraco SA, Grosjean F, Duval A, Rosa M, Lavanchy C, Ashok D, Haller S, Otten LA, Steiner QG, Descombes P, Luber CA, Meissner F, Mann M, Szeles L, Reith W, Acha-Orbea H (2012) Novel murine dendritic cell lines: a powerful auxiliary tool for dendritic cell research. Front Immunol 3:331. 10.3389/fimmu.2012.00331 23162549 10.3389/fimmu.2012.00331 PMC3491238

[30] Gargaro M et al (2022) Indoleamine 2,3-dioxygenase 1 activation in mature cDC1 promotes tolerogenic education of inflammatory cDC2 via metabolic communication. Immunity 55:1032-1050.e14. 10.1016/j.immuni.2022.05.003 35704993 10.1016/j.immuni.2022.05.013 PMC9220322

[31] Mezzasoma L, Antognelli C, Talesa VN (2017) A novel role for brain natriuretic peptide: inhibition of IL-1β secretion via downregulation of NF-kB/Erk 1/2 and NALP3/ASC/Caspase-1 activation in human THP-1 monocyte. Mediators Inflamm 2017:5858315. 10.1155/2017/5858315 28331244 10.1155/2017/5858315 PMC5346358

[32] Dowling JK, O’Neill LAJ (2012) Biochemical regulation of the inflammasome. Crit Rev Biochem Mol Biol 47:424–443. 10.3109/10409238.2012.694844 22681257 10.3109/10409238.2012.694844

[33] Erlich Z et al (2019) Macrophages, rather than DCs, are responsible for inflammasome activity in the GM-CSF BMDC model. Nat Immunol 20:397–406. 10.1038/s41590-019-0325-3 30742078 10.1038/s41590-019-0313-5

[34] Chung CP et al (2008) N-terminal pro-brain natriuretic peptide in systemic lupus erythematosus: relationship with inflammation, augmentation index, and coronary calcification. J Rheumatol 35:1314–1319 18528966 PMC2754266

[35] Westerterp M et al (2017) Cholesterol accumulation in dendritic cells links the inflammasome to acquired immunity. Cell Metab 25:1294-1304.e6. 10.1016/j.cmet.2017.05.017 28479366 10.1016/j.cmet.2017.04.005 PMC5514787

[36] Chen L et al (2020) MicroRNA-223-3p modulates dendritic cell function and ameliorates experimental autoimmune myocarditis by targeting the NLRP3 inflammasome. Mol Immunol 117:73–83. 10.1016/j.molimm.2019.10.024 31743855 10.1016/j.molimm.2019.10.027

[37] Inoue M, Williams KL, Gunn MD, Shinohara ML (2012) NLRP3 inflammasome induces chemotactic immune cell migration to the CNS in experimental autoimmune encephalomyelitis. Proc Natl Acad Sci USA 109:10480–10485. 10.1073/pnas.1201832109 22699511 10.1073/pnas.1201836109 PMC3387125

[38] Jankovic D et al (2013) The Nlrp3 inflammasome regulates acute graft-versus-host disease. J Exp Med 210:1899–1910. 10.1084/jem.20130355 23980097 10.1084/jem.20130084 PMC3782050

[39] Mak’Anyengo R et al (2018) Nlrp3-dependent IL-1β inhibits CD103+ dendritic cell differentiation in the gut. JCI Insight 3:e96322. 10.1172/jci.insight.96322 29515025 10.1172/jci.insight.96322 PMC5922280

